# Sample data processing in an additive and reproducible taxonomic workflow by using character data persistently linked to preserved individual specimens

**DOI:** 10.1093/database/bav094

**Published:** 2015-09-30

**Authors:** Norbert Kilian, Tilo Henning, Patrick Plitzner, Andreas Müller, Anton Güntsch, Ben C. Stöver, Kai F. Müller, Walter G. Berendsohn, Thomas Borsch

**Affiliations:** ^1^Botanic Garden and Botanical Museum Berlin-Dahlem, Dahlem Centre of Plant Sciences, Freie Universität Berlin, Königin-Luise-Str. 6–8, 14195 Berlin, Germany and; ^2^Institut für Evolution und Biodiversität und Botanischer Garten Münster, Westfälische Wilhelms-Universität Münster, Hüfferstr. 1, 48149 Münster, Germany

## Abstract

We present the model and implementation of a workflow that blazes a trail in systematic biology for the re-usability of character data (data on any kind of characters of pheno- and genotypes of organisms) and their additivity from specimen to taxon level. We take into account that any taxon characterization is based on a limited set of sampled individuals and characters, and that consequently any new individual and any new character may affect the recognition of biological entities and/or the subsequent delimitation and characterization of a taxon. Taxon concepts thus frequently change during the knowledge generation process in systematic biology. Structured character data are therefore not only needed for the knowledge generation process but also for easily adapting characterizations of taxa. We aim to facilitate the construction and reproducibility of taxon characterizations from structured character data of changing sample sets by establishing a stable and unambiguous association between each sampled individual and the data processed from it. Our workflow implementation uses the European Distributed Institute of Taxonomy Platform, a comprehensive taxonomic data management and publication environment to: (i) establish a reproducible connection between sampled individuals and all samples derived from them; (ii) stably link sample-based character data with the metadata of the respective samples; (iii) record and store structured specimen-based character data in formats allowing data exchange; (iv) reversibly assign sample metadata and character datasets to taxa in an editable classification and display them and (v) organize data exchange via standard exchange formats and enable the link between the character datasets and samples in research collections, ensuring high visibility and instant re-usability of the data. The workflow implemented will contribute to organizing the interface between phylogenetic analysis and revisionary taxonomic or monographic work.

**Database URL**: http://campanula.e-taxonomy.net/

## Introduction

**Fig. 1.**
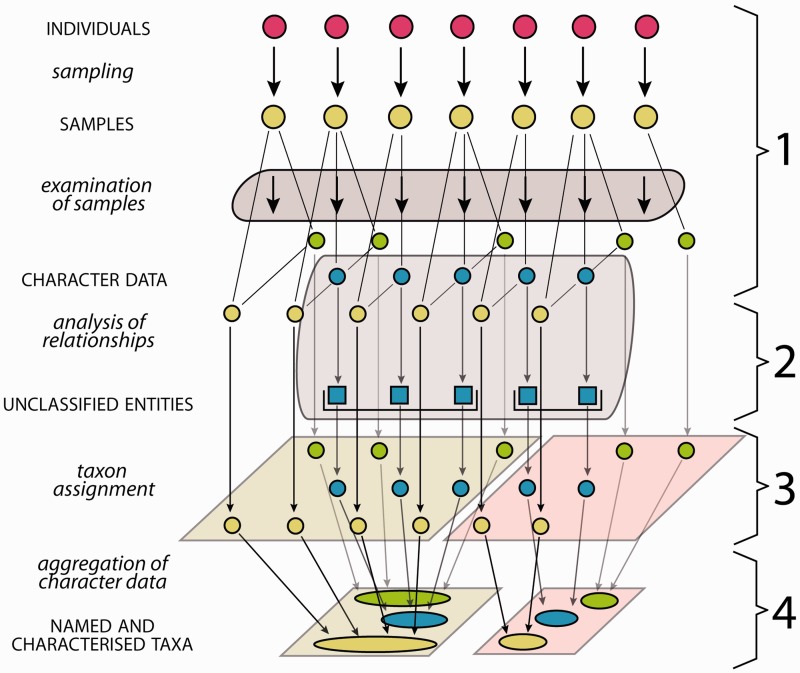
Generalized scheme of the steps in systematics from the investigation of organism individuals to the characterization of taxa. The first column lists the processes (lower case letters + italics) and products (upper case letters + normal style), the diagram illustrates the data flow and the last column numbers the steps as explained in the following: (1) samples of individuals are examined, providing different types of character data (green, blue, yellow), not all of them necessarily available for all samples. (2) Analysis of relationships (e.g. phylogenetic or tokogenetic), using e.g. available molecular character data (blue), reveals evolutionary relationships among the sampled individuals, grouping them into unclassified entities such as clades. In a phenetic approach, the evolutionary analysis in this step is replaced by an evaluation of morphological similarities and discontinuities. (3) In order to translate inferred (from whatever analysis) relationships into classification, the unclassified entities with the included samples and character data are assigned to taxa, also employing further character data types (yellow, green). (4) Naming of taxa and aggregating (summing up) of the character data from the individuals included results in named and characterized taxa. Further sampled individuals not included in the evolutionary analysis but matching the taxon characterization can be included, their data adding to the characterization.

Biological systematics, referred to as systematics in this study, aims to assess organismic diversity by attempting to identify natural biological entities above the individual level (taxa), to uncover their relationships and to characterize, classify and name them (1). All analyses in systematics (Figure 1) are based on ‘samples’, a term used in this study in the unspecified sense of a probe or examination object taken from an individual organism. Examination of these samples produces ‘character data’—often named ‘descriptive data’ (2, 3) and sometimes ‘comparative data’ (1)—a class of data referring to ‘taxonomic characters’ (4), which each have two or more states and can cover all data suitable to characterize a taxon in comparison with related or similar taxa. Character data that are suitable for use in evolutionary analyses are processed in order to group sampled individuals into natural biological entities. Evolutionary analyses may include to study tokogenetic relationships within a species, or to study sampled individuals as representatives of species in a phylogenetic context. The character data may be analysed also using a phenetic or other approach. The results in each case are initially unclassified entities, which in subsequent steps can be assigned to taxa and then be named (Figure 1) (5, 6). The taxon assignment of unclassified entities revealed from evolutionary analyses translates evolutionary relationships into a classification. This translation essentially employs decisions on appropriate circumscriptions and ranks of taxa, guided by certain sets of criteria, which may be subject to debate. Additional individuals that match these taxa can also be assigned to them. Taxon assignment of individuals, i.e. the process of matching sampled individuals with taxa, thus of their identification, uses a subset of character data as indicators that are considered diagnostic for a taxon and for its distinction from similar or related taxa. The available character data obtained from all sampled individuals of a taxon are finally ‘aggregated’, thus summed up, into a comprehensive ‘taxon characterization’ (7) (frequently but less appropriately referred to as ‘description’, see section two of this study). Taxon characterizations are thus the product of the taxon delimitation (5) and may vary in so far as different taxon delimitations are applied (‘taxon concepts’) (5, 8–10) or different geographic scopes may be considered. The characterizations of higher taxa (taxa that include subordinate taxa) are in the same way the product of taxon delimitation and are the sum of their included subordinate taxa. The taxon characterizations of all subordinate taxa making up a higher taxon are thus to be aggregated into the characterization of the corresponding higher taxon.

The generalized scheme in Figure 1 of steps from the investigation of organism individuals to the characterization of taxa also illustrates the interface between evolutionary analysis and taxonomy: both share step 1 (sampling and examination of samples), while step 2 (analysis of relationships) is the core domain of evolutionary analysis, and steps 3 and 4 are the core domains of taxonomy. If the evolutionary analysis in step 2 is replaced by an evaluation of morphological similarities and discontinuities, the result is a so-called ‘alpha-taxonomic’ classification. This article addresses the taxonomic part of that work process, thus step 1, and, taking up the results of evolutionary or other analyses from step 2, it also addresses steps 3 and 4. We are conscious of the fact that taxon characterizations of microorganisms and fungi may set different accents for the taxonomic work process (11).

Usually, a taxon characterization is based on the examination of a very limited set of individual representatives of the taxon and on a set of character data limited by the selection of examination methods applied. Consequently, any new, sampled individual as well as any new character data may affect the taxon characterization and/or the taxon delimitation. Moreover, in the vast majority of cases the evaluation of the sampled and examined individuals is still based just on morphological similarities and discontinuities (alpha-taxonomy) and remains to be confirmed by phylogenetic reconstructions. Actually, our understanding of the evolutionary history as well as the classification and naming of taxa necessarily is an iterative process, with an approximation to reality, often triggered by methodological innovations. The need for minor or major revisions or adjustments of established classifications and taxon characterizations, also affecting their names, is thus both pervasive and continuous (12–14).

The process of synthesizing our growing knowledge of biodiversity is challenging. Integrative taxonomic treatments in general, and monographs as a final product of systematics (15, 16) in particular, consequently represent the approximate knowledge at a given point of time. Societal demands for reliable, up-to-date, and authoritative products, such as biodiversity inventories, identification aids and encyclopaedic works on groups of organisms (17), call for name stability, while progress in systematics may affect established classifications and names.

One of the major problems involved is that print publications are too static to function as knowledge bases of organismic diversity. For this, biodiversity informatics has developed solutions to design synthesizing works in biodiversity research as dynamic ventures (18–22) and to facilitate data exchange by providing unified and convenient query mechanisms for distributed and often highly heterogeneous data repositories.

However, in order to organize dynamic approaches to such syntheses, they need to be generated from data that are structured in a standardized form and stored in an underlying database. Character data structured in character and state matrices (3, 4, 23) and data aggregation procedures for taxon-based character data are well established, although still applied by a limited number of workers. Several applications are available for storing structured taxon-based character data in order to generate identification keys and natural language descriptions, and to aggregate them from lower to higher taxa. Starting with the DELTA (DEscription Language for Taxonomy) (24) system (2) as the pioneer, others followed such as Lucid (25), Delta Access (26) and Xper2 (27). With the development of the XML-based SDD (Structure of Descriptive Data) standard (28), data in the DELTA and Nexus (29) standards (30) are becoming fully exchangeable, SDD compliance provided. With the NeXML exchange standard (31), recently an XML-based Nexus successor for representing taxa, phylogenetic trees, character matrices and associated metadata has been developed.

The implicit conclusions for the association between character data from sampled individuals and taxon characterization have, however, hitherto hardly been drawn with the necessary rigor. The Prometheus Model (3, 5, 32), an approach based on taxonomic working practices rather than on taxonomic outputs, is a remarkable exception. In order to make an investigation both transparent and reproducible, vouchers (commonly termed specimens) allowing an assured identification of the sampled individuals are permanently preserved. Consequently, the Prometheus Model emphasizes that the research process in systematic biology at the species level and below is specimen based, and the taxon characterization is the product of the included specimens, and the taxa above the species level are circumscribed by the subordinate taxa. The taxon characterization can thus only be determined and reproduced in an objective way by the included specimens. The Prometheus Model takes a specimen-oriented rather than a taxon-oriented approach. With the Prometheus Description Model, Pullan *et al.* (3) moreover perspicaciously addressed the need for the re-use and exchange of character data between different research projects, and modelled pioneering solutions for the main problems involved. This includes a solution for compatibility issues of character datasets from different sources and also the possibility of recording character data at various levels of concreteness, ranging from a single instance of a structure on a specimen to the individual specimen as such. Yet, the Prometheus Model was never developed to a tool available for taxonomic work.

Therefore, until today common taxonomic working practise is that the characterization of a taxon refers only collectively to a set of included specimens so that the character data are not associated with the individual specimens they were taken from. In this way, the only accurate way of achieving adjustments with respect to taxon delimitation and consequently to taxon characterization is the most laborious: re-examining the characters and specimens.

For a sound foundation of the character data aggregation procedure and in order to streamline taxon characterization, a reversible generation of a taxon characterization from the character data of the sampled individuals is necessary. The prerequisite for this foundation is to establish a persistent and unambiguous connection between each sampled individual and the data processed from it. Specimens remain the representatives of the sampled individuals after the conclusion of the systematic research process and are preserved and curated in corresponding research collections. The obvious conclusion should therefore be the establishment of an unambiguous association between the character states and ranges recorded for each specimen, or for each sample substantiated by a specimen, and their persistent connection with the specimen metadata. Any newly examined individual assigned to a certain taxon may then confirm or modify the taxon characterization upon re-aggregation of the character data. Once evolutionary analysis of character data reveals changes in taxon delimitations, its characterization can then be regenerated upon aggregation of the character data from the altered sample sets. The necessity to document the character data for the individual specimens rather than for taxa similarly applies to phylogenetic analyses, in particular for such based on morphological characters, where the corresponding problems have been clearly addressed (33).

This article presents the concept of a workflow and dataflow that blazes a trail in systematic biology for the re-usability of character data and their additivity from specimen to taxon level, and its implementation, using the EDIT (European Distributed Institute of Taxonomy) Platform (22). We first (part 2) explain our concept for the implementation of a persistent and unambiguous connection between character data and samples in the systematic research process. Subsequently (parts 3 and 4), we describe the implementation of the single steps of the workflow using the EDIT Platform.

Our solution aims to (i) establish a reproducible connection between sampled individuals and all types of samples derived from them during the research process; (ii) persistently link the metadata of all types of samples with the respective character data; (iii) record and store specimen-based phenotypic, geographic and environmental as well as molecular character data in formats suitable for data exchange; (iv) reversibly assign sample metadata and character datasets to taxa in an editable classification and display them and (v) organize the exchange of sample data sets via standard exchange formats. Finally, we discuss the opportunities that our solution opens up for the preservation of raw data and for the deposition of character datasets along with samples in research collections, and we identify fields where further developmental work is needed.

## Conceptual foundations of integrated sample data processing

### Organismic samples, their associations and data

In systematics, the analysed samples each directly or indirectly originate from a population of organisms in the field. Collecting samples of such a population creates a ‘gathering’ [the term is here used in the sense of the ‘International Code of Nomenclature for algae, fungi and plants’ (ICN)] (34, p. 156) for ‘a collection of one or more specimens made by the same collector(s) at one place and time’. The ‘gathering event’ (35) is thus connected to a specific time and location. The single gathering, to which usually a unique ‘field number’ or ‘collecting number’ is assigned, is a data object termed ‘field unit’ (35). We here use this term to refer to a single (named or unnamed) taxon, and either to a single individual, of which it may include one or more samples (depending on the size of the individual), or to a population, of which it consequently includes a number of individuals or parts of them. Therefore, the field unit can consist of one ‘specimen’ or a number of ‘specimens’ and in the latter case they are commonly considered duplicates of one another and are thus principally exchangeable with respect to their essential information content. This depends, of course, on the research context: population genetic analyses, e.g. require that duplicates must stem from the same individual. However, the concept of the ‘field unit’ also allows the handling of multitaxa gatherings; as taxon-ambiguous field units are permitted, it is, therefore, also applicable to the study of microorganisms.

**Fig. 2.**
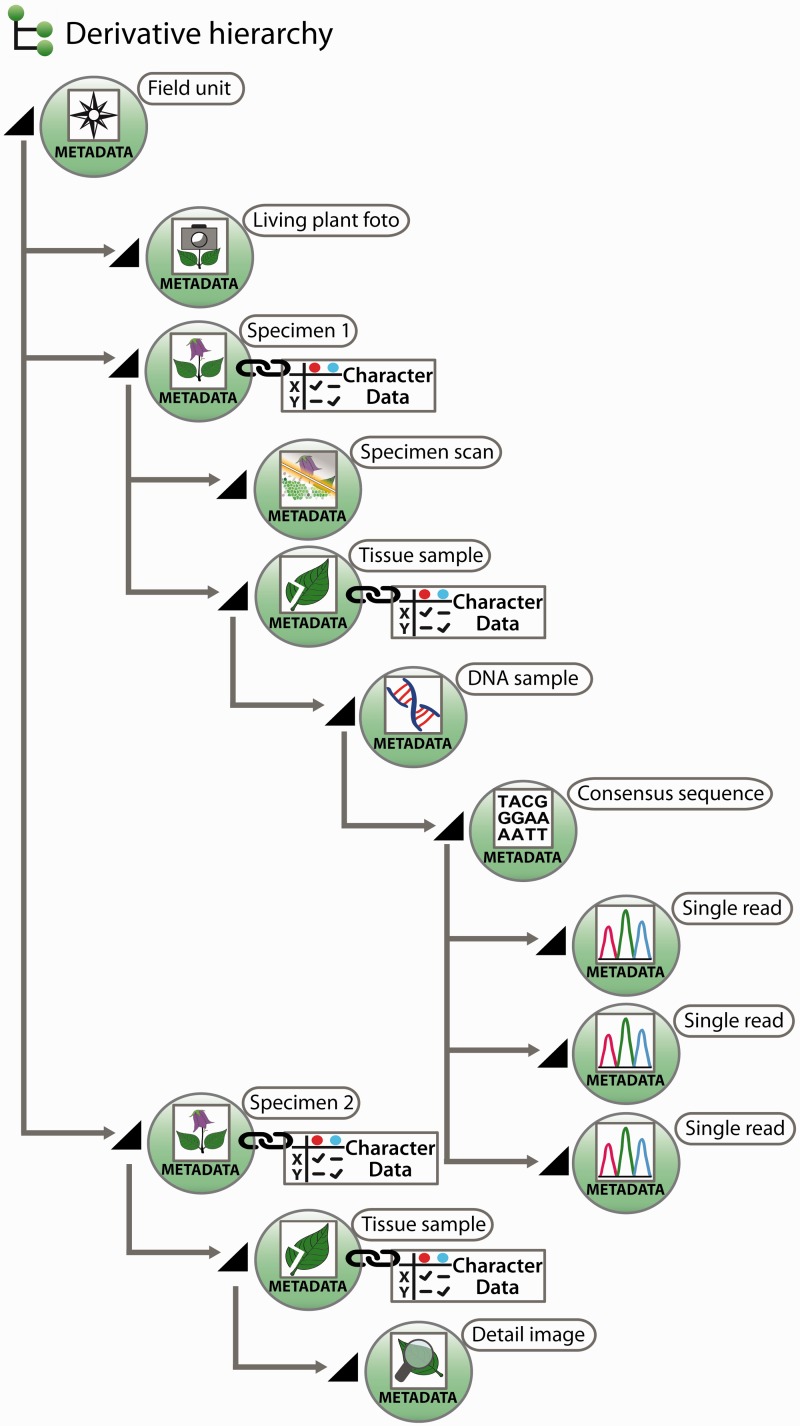
Exemplar scheme of samples with metadata and character data in a derivative hierarchy.

All further samples taken from a specimen of a field unit are termed ‘derivatives’, more precisely ‘specimen derivatives’ (‘derived units’) (35). Based on the field unit, derivation events can create a series of derivatives. Being products of derivation events, derivatives are usually hierarchically structured [e.g. specimen → pollen sample → scanning electron microscope (SEM) micrographs]. Both the derivative hierarchy and any single derivative are rooted to the field unit, ensuring that each derivative is rooted even if an intermediate derivative is lost, of ephemeral nature or has never been recorded. A first derivation step from a taxon-specific field unit is the individualization of specimens, the specimen thus constitutes a first derivative of the field unit (Figure 2).

The taxon assignment of a taxon-specific field unit is normally inherited (in terms of data processing) to and valid for all the field unit’s derivatives. Similarly, a taxon assignment to a derivative is inherited to all its other elements and the field unit. Erroneous assignments of samples to a taxon-specific field unit may result from misidentification in the field or in light of novel insights following later analyses leading to the re-circumscription of a taxon. Another possibility is the consideration of a taxon which was outside the scope of the original gathering (e.g. epiphytic lichens, parasites) (36). Consequently, such samples need to be separated (at least in their virtual representation) and assigned to the correct newly developed taxon-specific field units.

Once a derivative becomes part of a collection (e.g. a herbarium), and thus a collection object, a metadata type termed ‘collection unit’ can be assigned to the derivative.

Any sample that is examined, regardless of whether it is newly collected in the course of the research process or taken from a research collection, which, in the latter case, may be from a living collection (e.g. botanical or zoological garden) or museum collection, is assigned to a specimen derivative hierarchical level. Two types of data are principally associated with each sample:

- ‘Sample metadata’ predominantly include the event-related information, including sample origin, collecting locality, observations in the field, gathering method, preparation process, derivation events in the examination, position in the derivative hierarchy, accession and storage place in a collection and more. The main functions of the sample metadata are to give the sample a unit identity and to make it reproducible or at least traceable. The core of the sample metadata is found on labels attached to a collection object, which may be supplemented, in the case of poorly labelled ‘historical’ specimens, by data from related sources, such as published reports on expeditions and laboratory protocols. The ‘taxon assignment data’ are a particular type of sample metadata, which indicate the taxonomic identification of a (taxon-specific) field unit and all its derivatives, including the taxon name, typification, name of identifying scientist, date of determination, synonymizations and determination history. The taxon name connects the sample and its data to a certain taxon in the classification. One type of sample metadata has a double nature: data related to the gathering event in the field, such as locality data, gathering date and observations on the gathered organism, will also contribute to the characterization of that taxon (described in detail below), by information such as distribution, ecology or phenology.
- ‘Character data’ include all primary (raw) and secondary (edited or derived) data gained through the examination of a sample. They can theoretically comprise the entire phenome (the entirety of a taxon’s ‘traits’ or ‘features’), genome information plus all related geographical and environmental data. If character data have an unambiguous connection to a single documented sample, they are referred to as specimen-based as opposed to merely taxon-based character data. ‘Structured character data’ are organized in a matrix distinguishing characters and two or more states, in contrast to ‘textual character data’ (e.g. in a natural language description).

The term ‘trait’ is conceptually narrower than ‘character data’. Trait refers to phenotypic variation relative to genetic and environmental factors for particular phenotypes. However, it has been used ambiguously either corresponding with a character or, more commonly, with a state. The definition of the term trait has been widened in ecology to functional and physiological traits. The term character data is inclusive of these as well. The terms ‘descriptive data’ (3, 27) and ‘comparative data’ (1) are largely synonymous to character data. However, the former in particular has often been used in the narrow sense, referring to the data of the ‘taxon description’, which historically ranges from a brief morphological differential diagnosis to a more or less comprehensive morphological description of a taxon. The term ‘factual data’ (37), coined in the context of modelling data relations of taxon concepts and names, is wider than the above mentioned terms. It refers to any factual information that is connected to a taxon and thus also includes information about human uses or the conservation status of a taxon, which is too extensive to be included with the character data.

Derivation events frequently lead to samples that are either not preserved as physical objects, or they lose their physical concreteness and then are merely present as digital objects. Examples include the SEM analysis of pollen samples, where only the digital SEM micrographs remain, or the amplification and sequencing of markers from a DNA isolate, where after an isolate has been used only the trace files remain. Where derivation events transform physical into digital objects, the digital objects can, with similar justification, be treated as sample derivatives or as data gained from samples. Generalizing this, one could consider the generation of character data from a sample as a derivation event, and the obtained character dataset as a further derivative instead of a sample-based characterization item. We have decided, however, to treat in the data model only derivatives in the narrower sense, i.e. not character data as derivatives, but in the interest of user convenience, a joint visualization of derivatives and character datasets in the user interface independent of the model decision is possible (Figure 2, and see below).

### Processing sample metadata

Usually, samples in a research project in systematic biology are to some part newly collected, while to some other part obtained from research collections, either as physical objects or as digital representations. A required functionality is therefore the communication with research collection databases or corresponding aggregators to search for and to import digital sample representations and sample metadatasets. The standard exchange formats ABCD (Access to Biological Collection Data) (38) and Darwin Core (39) should be supported.

Imported metadatasets may need, at some stage, to be edited. Editing may include the following: (i) completion of label data; (ii) addition of relevant metadata from other sources, such as duplicate samples and itineraries, for insufficiently labelled historical collection items; (iii) standardizations, such as making collector names unambiguous and conversion of data into standard units; (iv) completion or correction of the parsing of the metadata into the relevant data fields; (v) clarification of toponyms and georeferencing localities and (vi) fixed associations of taxon names with specimens following nomenclatural typification. Editing with respect to (iii), (iv) and (v) is essential for the processing of metadata elements in the context of taxon characterization, such as georeferenced localities for distribution mapping or collecting dates for phenology. Type information (vi) is to be processed in order to fix the application of a name to the taxon containing this specimen.

In the case of collection items or their derivatives for which no digital metadatasets are available, these need to be newly entered into the data store. In the case of newly collected material for an investigation, it depends on institutional workflows; the material may be first accessioned by the research collection and its metadata can then be imported from the institutional collection database, or vice versa. Exporting the newly entered and the edited sample metadatasets to institutional research collections is possible using standard exchange formats ABCD (38) and Darwin Core (39). Furthermore, this can be done in a way that clearly distinguishes original and edited data.

### Linking specimen-based character data to sample metadatasets

Sample examination produces specimen-based character datasets of various types and formats. These datasets are characterization items to be persistently linked to the analysed samples (represented by their metadataset) and via the derivative hierarchy also to the individual specimens documenting the individual organismic source of these data. For all stages of the research process the corresponding character datasets should be available, visible and easily accessible. An export of the sample metadatasets to the respective research collection should contain a stable link to the existing character datasets, or even be directly associated with the available character datasets.

### Taxon assignment of samples and their data

Through assignment to a taxon, the field unit as the root of the specimen derivative hierarchy becomes connected to the taxonomic classification of a group of organisms. As a consequence, all connected derivatives, the sample metadata corresponding to the gathering and the character data resulting from the examination of a sample also become assigned to that taxon. The taxon assignment is thus effective for all levels in the derivative hierarchy and is reversible. Samples and character datasets assigned to taxa should be easily visible and accessible.

Simple moving of a taxon within a classification or renaming does not affect the connection between samples and taxa. In contrast, re-delimitation of a taxon, which involves a re-evaluation of the included samples and/or character data, will also demand to adjust the taxon assignment of the samples.

### Aggregating specimen-based character data at the taxon level

The essential procedure for any taxon characterization is the aggregation of the specimen-based character data to taxon character data according to the delimitation of the taxon. The extent and type of the aggregation depends on the data type and structure, and the means and purposes of their use at the taxon level. This may include an ‘appending aggregation’ (leaving the appended data unchanged), such as DNA sequence data, or a ‘merging aggregation’ (statistical values), such as the measurement of floral features or altitudinal distribution ranges.

It is necessary for data aggregation to be designed as an iterative and automated procedure, permitting changes in the sample basis of the data, due to changing taxon delimitation or data availability. This would trigger a new round of aggregation, which replaces the results of the preceding one. The prerequisites are that the data are structured and compatible. Taking the domain of morphological data as an example, it becomes evident that the main obstacle is to ensure that sets of characters and states are compatible during specimen investigation across a larger group of organisms. Aggregation for distant taxa of the same larger group of specimen-based data at the lowest taxon rank applied must not use incompatible matrices in order for subsequent aggregations at higher ranks to be successful.

A number of applications exist to create taxon-based character and state matrices and to further process them for the generation of identification keys and natural language descriptions, and to aggregate them from lower to higher taxa (2, 25–27). As long as compliance with the XML-based SDD standard (28) is provided, the data are exchangeable between the applications. Problems regarding exchangeability of structured data matrices, term ontologies including addressing homology issues and the character data model (3) remain to be addressed in future work.

Fortunately, the aggregation of character data from lower to higher taxa is principally the same as the primary aggregation of specimen-based character data at the taxon level, with respect to data structure and aggregation algorithms. The same applications can thus be employed in order to record and aggregate specimen-based character data.

## Workflow implementation using the EDIT Platform

### Extending the EDIT Platform to handle the variety of sample data

Our concept for an integrated workflow for sample data spans from the selection of sampled individuals to the aggregation of character data for named taxa (Figure 1), but intentionally it excludes the capacity to conduct evolutionary analysis of sampled individuals. However, it aims to include the entire data recording for the examined samples (metadata and character data) and to hold and provide the specimen-based structured character data (morphological and molecular) of the sampled individuals for any evolutionary analysis, such as phylogenetic reconstruction. The datasets for the sampled individuals can be assigned to taxa according to the results of the analysis and the character data can be aggregated to add to the taxon characterization.

The implementation of this workflow requires a web-enabled working platform, readily allowing networking of distributed team workers, capable of the pertinent data exchange standards for collection data, with suitable interfaces to handle character data, and capable to handle taxonomic classifications. Therefore, the ‘EDIT Platform for Cybertaxonomy’ (22, 40, 41), or shorter, ‘EDIT Platform’ has been selected for development of our workflow model. The EDIT Platform provides the necessary basic functionalities which require minimal extensions, especially in the specimen module. The EDIT Platform is based on the ‘Common Data Model’ (CDM) (42), which is a comprehensive object-oriented taxonomic information model covering the flow of taxonomic information from fieldwork to data publication. The pivot of this model is the ‘taxonomic concept’ (or ‘potential taxon’) being strictly separated from scientific names. This approach was originally developed by Berendsohn (43) and later refined and implemented in the ‘Berlin Model’ e-Platform (44, 45). Added to this was a rule-based ‘transmission engine’ for the transfer of character and other taxon-related ‘factual data’ between concepts in a network of taxonomic concepts (46, 47). The CDM complies to the relevant data standards of biodiversity informatics (Biodiversity Information Standards [TDWG], also known as Taxonomic Databases Working Group) (48), including ABCD (38), Taxon Concept Schema (49), SDD (28) and Darwin Core (39). Besides the EDIT Platform it is also the basis for Creating a Taxonomic E-Science (19).

An outstanding feature of the EDIT Platform is its connectivity and interoperability among the emerging international biodiversity informatics infrastructures through standardized web service layers. Data exchange interfaces to various biodiversity e-infrastructures have been implemented including the GBIF (Global Biodiversity Information Facility) Checklist Bank (50), Biowikifarm (51), Scratchpads (52), Plazi (53), BioVeL (54) and Biodiversity Heritage Library (55).

The EDIT Platform is open source and applicable to all groups of organism, in particular those covered by the ICN (34) and the International Code of Zoological Nomenclature (56). Current applications are monographic in approach (Cichorieae Portal; CLD-CoW Portal; Palmweb) (57–59), regional checklists (60) or floras (61).

### Basic functionalities of the EDIT Platform, scalability and use cases

The EDIT Platform can be employed to handle and connect the different data types associated with the samples right from the start of a research process in systematic biology. It provides three main components:

- Data repository and server: The CDM store hosts the taxonomic classification, the metadata and character data for samples, and also links to external web resources. All data objects are accessible through Java and web service interfaces.
- Taxonomic Editor: The core application of the working platform functionality is the Taxonomic Editor. Among others, it allows the searching for, importing, entering and editing of all taxon- and specimen-related information stored in the CDM.
- Data Portal: The portal provides a dynamic visual user interface for online publication. It gives access to all publication-relevant data objects stored in the CDM. Classifications are represented by a taxon tree, which allows users to navigate through multiple hierarchies. The portal links out to biodiversity e-infrastructures such as BHL (Biodiversity Heritage Library) (55) and GBIF (50) and has advanced functions for visualizing species distributions and multimedia files.

Although the EDIT Platform is being designed to support the distributed research process in systematic biology from sample acquirement to the publication of a monograph, more frequent use cases are taxonomic revisions or phylogenetic analysis of smaller groups of organisms, and in some cases a combination of both. Such work is frequently conducted by an individual scientist or a working group and usually without a long-term dedication to a particular group of organisms. Cases like these often lack the active institutional support, in particular IT infrastructure. Instead of the fully operational ‘community installation’, they may use the easy to install ‘individual installation’, which allows a single worker to edit and maintain an individual dataset on a personal desktop, and a ‘group installation’ for a working group with a shared data repository within an institutional intranet. In contrast to the individual installation scheme, the group installation comes with a data portal to publish the data electronically (http://cybertaxonomy.eu/cdm-setups/). An installation with the full implementation of the workflow described in this article is expected to be available for download by the end of the project in December 2015.

## Steps of the integrated sample data workflow

### Scope of the workflow

Here we outline the steps of the integrative processing of sample metadata, sample character data and their taxon assignment, as it has been developed and is being implemented in the EDIT Platform. Its aim is to create the prerequisites for a consistently specimen-based research process in systematic biology. This includes the following: (i) establishing a reproducible connection between sampled individuals and all types of samples derived from them, which allows instant sample metadata processing, including de novo input, retrieval, import, documented (for potential synchronization with external sources) editing, display and export, within the research process; (ii) stably linking the metadata of all sample types with the respective character data gathered from them, by providing means for handling specimen-based character data (morphological and molecular) and for firmly linking them to the sampled individual; (iii) recording structured specimen-based character data in formats allowing data exchange and easy retrieval; (iv) reversibly assign sample metadata and character datasets to taxa in an editable classification, allowing optional publication of the investigated samples in the context of taxon-based information portals and (v) organizing data exchange via standard exchange formats and enabling persistent, specimen-linked storage effectively accessible for humans and machines in research collections, ensuring high visibility and instant re-usability of the data.

The workflow described is still a work-in-progress. Although the foundations were laid for the implementation of the entire workflow in the EDIT Platform, its single steps have been elaborated so far to different depths. It will be workable throughout by the end of 2015 but in particular the handling of structured (morphological) character data will have to be considerably improved to meet all essential needs by a corresponding follow-up project proposal submitted to the German Research Foundation.

### Establishing a reproducible connection between sampled individuals and all types of samples derived from them

#### Searching, retrieving and importing of sample metadata

**Fig. 3.**
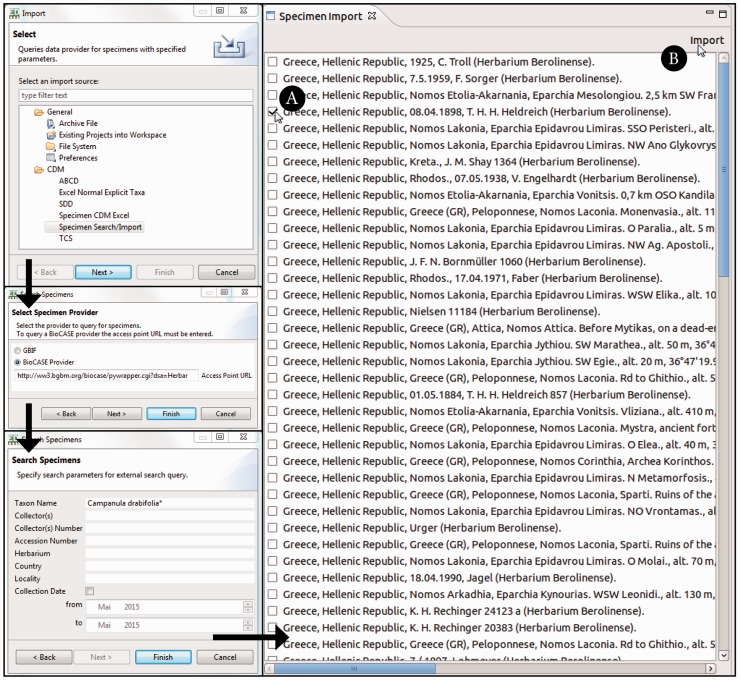
Taxonomic Editor of the EDIT Platform, derivatives perspective: screenshot of the specimen query and import interface. The black arrows indicate the single menu steps that specify the import. After the import form has been sent out, the search results are listed in a separate tab. The specimen can then be chosen (**A**) and the import of the datasets can be completed (**B**).

The ‘specimen search’ in the Taxonomic Editor is defined by the search parameters and the query interface supported by a specimen data provider. The implemented system supports a list of specimen data providers and allows users to decide which provider to query. It converts the query to the required format for the provider’s interface. One option currently implemented is GBIF (50), which is queried via web services; the other option is BioCASE (62), the providers of which are queried with a specific XML-based query protocol (63) (Figure 3). Common search parameters are taxon name, collector, collector’s number and country. Specifying two or three of these is usually sufficient to reduce the search results.

The Taxonomic Editor provides an import routine that can both convert the different formats returned (ABCD and Darwin Core) to display the results in a CDM-unique, standardized format and provide the functionality to store the specimen data in the CDM, merging it with existing data. The imported data are stored with the provider’s original unique identifiers to enable data synchronization.

#### Editing metadatasets

The specimen module of the Taxonomic Editor has been extended to provide full user interface functionality for displaying and editing all levels of the derivative hierarchy. The tissue and molecular sample modules of the CDM have been extended to enable full data coverage. Fields with pre-defined or user-defined elements have been selected to avoid redundancy and ensure coherent use of terms and names, e.g. for primers and DNA markers.

#### Building and editing specimen derivative hierarchies

**Fig. 4.**
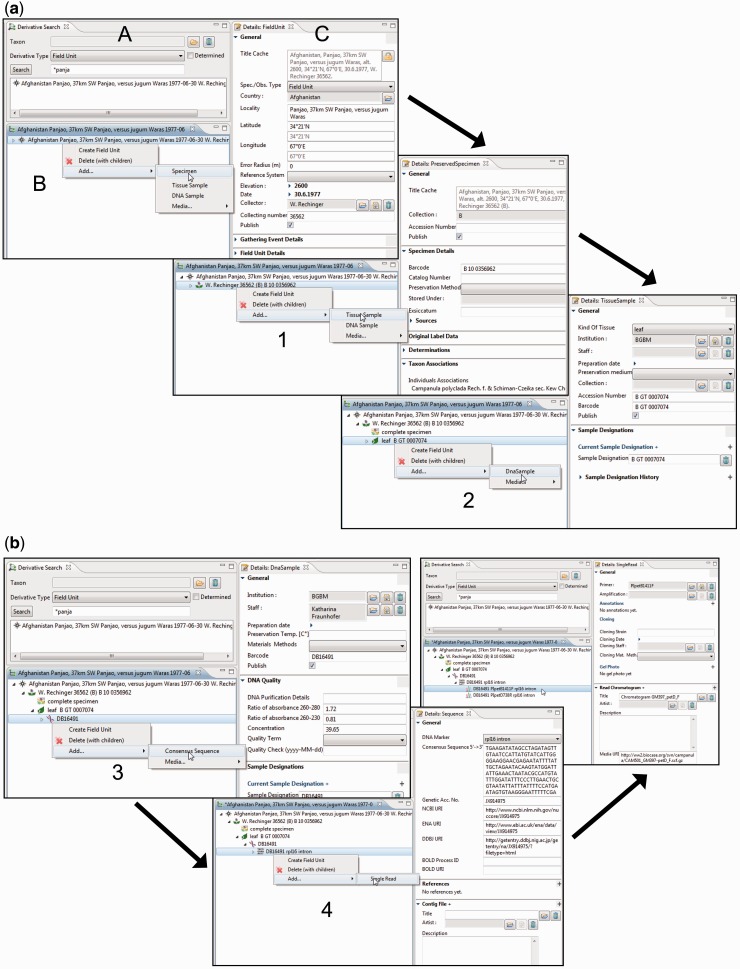
Taxonomic Editor of the EDIT Platform, derivatives perspective: screenshot of the derivative view displaying the derivative search (A), the derivative hierarchy (B) and a details view for the corresponding metadata (C). Screenshots illustrate the stepwise establishment of a derivative hierarchy by successive creation of derivatives and insertion of their data: (**a**) addition (1) of a tissue sample and (2) of a DNA sample; (**b**) addition (3) of a consensus sequence with links to one of the INSDC (International Nucleotide Sequence Database Collaboration) databases, (4) of single reads (Sanger sequencing trace files) and/or a contig file.

The derivative hierarchy is displayed as a tree in a separate interface, the ‘derivative search view’ (Figure 4A). ‘Derivative view’ (Figure 4B) and ‘details view’ (Figure 4C) form a functional unit that allows the convenient access to, and the creation and processing of, derivatives and their data. The field unit element is obligatory because it is the root of the derivative hierarchy and appears, if not manually created, automatically once a specimen or any other sample is entered or imported. All subsequent derivation steps and derivative types are prearranged in a hierarchical order according to the typical research workflow.

According to our concept of the derivative hierarchy, the derivative view holds a central position in the specimen module of the Taxonomic Editor. It is used to build the derivative hierarchy, thus to select derivatives, to visualize associated character datasets of the respective samples, to add and edit sample metadata and to display the hierarchy with all its data types in the Taxonomic Editor as they may also appear in the Data Portal. An example of such a prearranged derivative hierarchy (Figure 2) in the Taxonomic Editor is as follows: field unit →specimen collected → tissue sample taken → DNA isolated → DNA trace file created by the sequencer → consensus sequence generated from the contigs (Figure 4). In all such cases, the full sequence of the derivatives is not mandatory and can be applied as appropriate. For example, if no tissue sample and DNA isolate are stored, the trace file or consensus sequence can directly be attached to the specimen.

By selecting the details view, input options are provided for the essential metadata of each derivative. In the case of molecular data, the necessary terms and input options are matched with those compiled for the GGBN (Global Genome Biodiversity Network) network (64). The full extent of data covered by other repositories can be accessed via links in the details views (Figure 4B).

#### Versioning, synchronizing and exchanging metadatasets

**Fig. 5.**
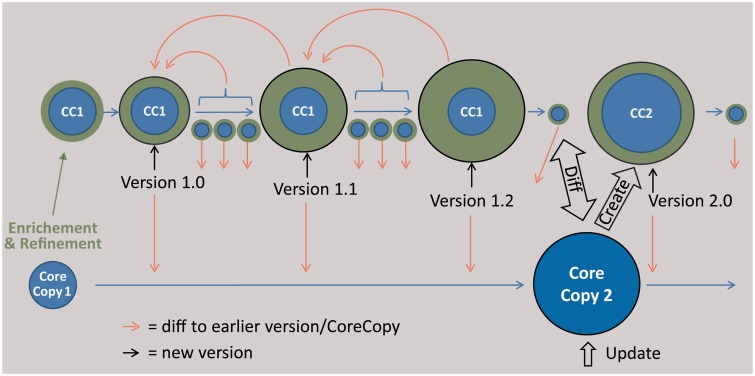
Scheme of the envisaged versioning functionality for sample metadata. The core copy is a copy of an imported dataset of an external provider, which is edited (green data). The versioning support of the CDM database, reporting every single change in the data, is used at certain intervals to create versions of the data, which can be compared using a diff viewer. The result of a subsequent query at the provider is stored as a new core copy, which can be compared with the latest version based on the first core copy and subsequently be edited.

The editing process (adding, deleting or changing data) will enrich and refine the original metadataset. These changes are separated from the original dataset, resulting in two semantic parts of sample metadata: (i) the ‘core copy’, the original dataset from an external provider and (ii) ‘enrichment and refinement’, the edited data which can be subject to a manual versioning by employing the auditing functionality of the EDIT Platform (Figure 5). On this basis, a special ‘Diff-Viewer’ can be implemented in the future to visualize the differences between versions and additionally allow the user to revert changes to an older version (Figure 5). Edited and newly entered specimen metadatasets can be provided to the corresponding research collections using AnnoSys (65) as a back-end service for storing and communicating the annotations. AnnoSys provides the functionality to annotate publicly displayed specimen records by users, to keep track of, and to inform data providers about annotations. AnnoSys exposes the annotations in the ABCD standard exchange format. The exposed dataset will include the documentation of the editing of the core copy to give the providers the opportunity to update their data. Conversely, the researchers can ask providers for a possible update of an earlier imported core copy and manually update their local copy.

### Stably linking character datasets to the sample derivative hierarchy

The central interface for linking specimen-based character datasets to the sample derivative hierarchy is the ‘factual data view’ of the Taxonomic Editor. The addition of such character data is displayed in the derivative hierarchy, where the derivative symbol is then replaced by a ‘derivative + character data’ symbol (Figure 2). Storage of the sample derivative hierarchy data in the CDM is configured to include the information about and, optionally, a stable link to external character datasets, or the stored character datasets themselves.

### Recording and storing specimen-based morphological and molecular character data

#### Storage

Specimen-based character data can be stored and curated in the EDIT Platform using the Taxonomic Editor, independent of their format. Data available in files from external applications can be stored and linked via the Web. For storing data files of various types in a working environment, we are using a server with Apache Subversion (svn, https://subversion.apache.org/), which combines convenient accessing of the file repository (e.g. using TortoiseSVN) with the advantages of a versioning and revision control system. The files are publicly available via a URI (uniform resource identifier). Mere textual data can be stored in free text fields of the CDM data store, some types of structured data can be directly mapped to the corresponding CDM classes for structured factual data. A fully functional data management, however, requires structured data in the supported exchange formats (see below).

For recording and editing character datasets, the Taxonomic Editor provides the ‘Factual Data View’ and specialized views for different data types, in which seamless integrations of otherwise independent applications are operational.

#### Structured morphological character data

For the recording and processing of structured morphological and related types of character data, the Xper^2^ software (27) is used. This software enables free creation of matrices of characters and character states and the recording of qualitative and quantitative character data of specimens and derivatives. In a recent paper (6, p. 295–296) we have outlined an approach, employing a terminology server and semantic web technology to ensure the compatibility of characters and states taken across a larger group of organisms, which we identified as a main challenge in part 3, above. There, we have also proposed a strategy as to how the wealth of unstructured textual descriptions in the literature can, in a controlled way, be employed in the frame of an otherwise specimen-based approach relying on structured data for taxon characterizations at lower taxonomic rank. Implementation of these approaches is subject to a corresponding follow-up project proposed.

#### Molecular character data

For recording and processing molecular (DNA) data, the Taxonomic Editor has been extended using several GUI (graphical user interface) components that display pherograms (trace files from Sanger sequencing) imported from AB1 or SCF files with their base call sequences and allows the combination of these in contig alignments and the creation of consensus sequences. The user can easily manually correct the base calls or edit the contig alignment and the consensus sequences. To achieve this, a new open source Java library called LibrAlign (66) has been developed. It provides powerful and flexible GUI components for displaying and editing raw data and metadata for sequences and alignments. Although LibrAlign was mainly developed for use in the Taxonomic Editor, its components have been designed to be of general use for other developers in the scientific community and it may be integrated into any Java GUI application, based on Swing, SWT, Eclipse RCP and Bioclipse (67), and it is interoperable with the CDM Library (42) and BioJava API (application programming interface) (68). Furthermore, support for importing and exporting whole contig alignments in various formats, such as FASTA, Nexus (29), MEGA (69) or NeXML (31), is currently implemented using JPhyloIO (70) in combination with LibrAlign. JPhyloIO is another general purpose Java Library developed for the Taxonomic Editor that provides event-based format-independent access to different sequence and alignment file formats. It is closely integrated with LibrAlign, but can also be used in the development of any application that does not use LibrAlign.

### Taxon assignment of sample metadata and character datasets

#### Adding sample data to a classification

The classification used for an investigated group of organisms can be displayed and edited in the ‘taxonomic perspective’ of the Taxonomic Editor, a pre-defined and pre-ordered set of graphical interfaces. This enables taxonomic hierarchies with synonymies to be imported, created and edited, including complex re-classification operations. The taxon assignment of a specimen or derivative hierarchy is effected in the details views of the derivative view or in the factual data view, where a taxon of the stored classification can be selected (and deselected). In this way, the derivative hierarchy with all linked character data becomes assigned to a certain taxon. If the status or position of a taxon is changed during the revision of a taxonomic classification in the taxonomic perspective of the Editor, all appended sample metadata and character data remain with the taxon. If one taxon is united with another one, the appended sample metadata and character data are synchronously moved with the taxon and their former placement is recorded. If a changed circumscription of a taxon requires the moving of specimens to another taxon, their former placement also is recorded.

#### Aggregating specimen-based character data at the taxon level

Iterative character data aggregation procedures are being implemented in the EDIT Platform for two different data types.

- Occurrence data: primary aggregation of geographical coordinates will result in dot distribution maps in the Data Portal. Aggregation of combined area unit distribution and occurrence status data at the same or from lower to higher taxon ranks is currently operated using a corresponding transmission engine. This rule-based engine aggregates distribution information (including occurrence status data) for a given taxon and region, recursively using its subtaxa and subregions. In the case of conflicting status values, decisions are made on the basis of defined priority rules.
- Character data stored in SDD-compliant character-state-matrices: the Xper^2^ software for character data management and interactive identification (27), which is integrated into the EDIT Platform, provides algorithms for data aggregation, merging numerical data while appending categorical data. The primary aggregation of the specimen-based character data at taxon rank currently only tentatively allows the automated generation of a natural language taxon description from the matrix. However, a workaround is the manual editing of the data using a description template. The storage of structured character data also enables the use of the data matrix for interactive taxon identification with the aid of multi access keys accessible through the data portal’s Keys Tab which we describe below.

#### Publishing sample metadata and character data with the CDM Data Portal

**Fig. 6.**
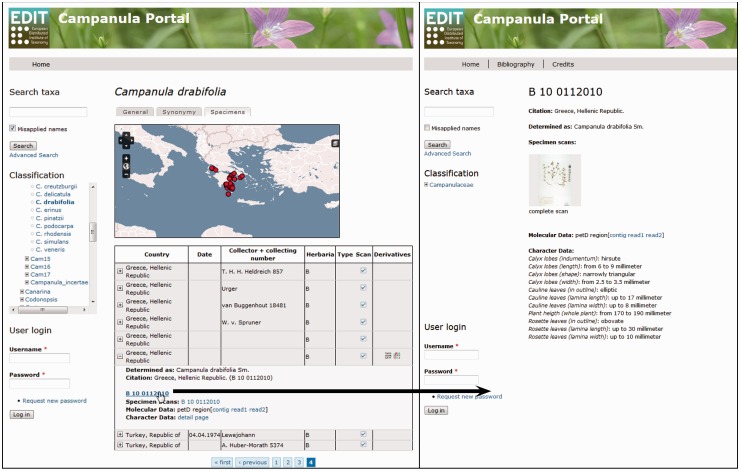
Data Portal of the EDIT Platform: screenshot of the *Campanula* data portal displaying the specimen tab visualizing the specimens and their derivatives available for a taxon. The Derivatives column indicates availability of additional datasets by displaying the respective icons. Clicking on a row (A) folds out the table cell and the listed items (here, specimen scan, DNA sequence contig and trace files) can be accessed by following the links given. The specimen ID functions as a link (B) to a separate specimen page where all derivatives of this specimen are clearly arranged, character datasets are provided and respective files are linked; clicking on the specimen scan thumbnail (C) opens the specimen scan in a separate browser window.

The EDIT Platform, unless in the individual installation of the software, allows the visualization of the data through its online Data Portal, which is customizable in its basic structure according to one of the principal aims: (i) a systematic revision or monograph providing maximum data, (ii) a flora or (iii) a checklist with the most restricted array provided. Classifications and taxon-related data are visualized in the portal and are accessible through a navigable taxon tree or via taxon name, area and subject searches. A data portal with the function of a systematic revision or monograph presents the information for each taxon, independent of its rank, in five basic tabs: (i) the ‘general tab’ displays the summarized taxon-based character data organized in feature chapters; (ii) the ‘synonymy tab’ displays the detailed synonymy and typification data organized in blocks of homotypic synonyms; (iii) the ‘image tab’ displays stored images; (iv) the ‘key tab’ offers identification keys (interactive or single access) optionally for taxa including subordinate taxa and (v) the ‘specimen tab’ finally displays the investigated or determined specimens with their derivative hierarchies and available character datasets, as well as a dot distribution map for the taxon based on the georeferenced specimens (Figure 6). Setting the ‘publis’ flag in the Taxonomic Editor for a specimen derivative hierarchy and the appended character datasets displays these data in the Data Portal. A search function, still in preparation, will allow users to filter certain derivative types and their data in the specimen tab of the Data Portal. For each specimen and its derivatives besides the expanded table view, a separate page with metadata, character datasets and links to other available character datasets can be opened (Figure 6). The *Campanula* Portal (71) (see, under ‘Preview’ on the ‘Welcome’ page, the exemplar taxa listed) is being used to visualize exemplars of taxa with various types of specimen-based datasets.

Using the publication services of the EDIT Platform, more specific outputs can be designed for publication of subsets of data in print or electronic publication media.

### Data exchange via standard exchange formats and enabling persistent, specimen-linked storage in research collections

Exchange of sample metadata between the EDIT Platform, research collections, biodiversity networks and collaborators is managed using ABCD (38) and Darwin Core (39) as the standard exchange formats. Both formats allow the import and export of the combined sample metadatasets of entire derivative hierarchies, such as represented, e.g. by the specimen with its scan and tissue sample collection for DNA extraction. Moreover, data import of such a derivative hierarchy further extended for isolated DNA sources plus marker consensus sequences with their contig files and corresponding pherograms has successfully been tested from the GGBN network (64) to the CDM Platform. Even our still further reaching concept of persistently and stably linking morphological and other types of character data with sample metadata and combined sample metadatasets of entire derivative hierarchies is already possible. ABCD currently offers a container element (‘<MeasurementsOrFacts>’), which can be used as a workaround to store atomized data, a complete character data matrix or a link to such a matrix. In this way, the exchange of the sample metadata with the respective research collection can include the information about and, optionally, a stable link to existing character datasets, or even the stored character datasets themselves. In the proposed follow up project and in connection with the development of ABCD 3.0, we envisage a more straightforward implementation for the exchange of associated structured character datasets. This will lay the foundation to popularize the association of (structured) character data with sample metadata, as well as their display and effective accessibility for humans and machines in research collections, ensuring high visibility and instant re-usability of character data through research collections.

## Perspectives

Our solution emphasizes the editing and enrichment of specimen metadata (e.g. taxon identifications, nomenclatural type status designations, georeferencing) by the researchers in the course of their examination of the material, as well as on the synchronization of edited data with the existing datasets. Doing so, it takes into account that the rapid advancements in the digitization of research collections have conducted the work and data flows related to collections in an analogous and a digital branch. Consequently, solutions have to be designed for the various use cases to ensure that revised and enriched metadatasets can conveniently be connected to the collections (65, 72).

Moreover, our solution, which streamlines the taxon characterization through establishing a persistent unambiguous relation between each sampled individual and the corresponding data, also opens new opportunities for the old problem of securing raw data associated with the research process in systematic biology. Primary research data do not only include pure data but also digital representations of preparations from specimens, ranging from light or scanning electron micrographs to sequencing trace files. Currently, if specimen-based character data are recorded, these are frequently treated as raw data, not usually included in publications, or, e.g. micrographs, published in a very limited selection only. At best they have, in more recent times, been deposited in repositories (73–75), otherwise they are still frequently considered only worth short-term preservation and disposed after the compulsory periods of record keeping, if not earlier (76). The deeper reason for not preserving raw data is often the lack of appropriate means to document, persistently link and visibly store them. Additionally, individual research databases are often not integrated in institutional data management strategies (77). National research funding bodies increasingly recognize the need for permanent storage facilities for primary research data (75, 78). However, the investment of extra work for long-term storage of specimen-based character data in a meaningful way is not economic as long as their re-use is not well organized. Primary research data therefore must appear effectively visible in a potential use context, must be technically compatible and so on. Evidently, the mere presence of data in some sort of public repository does not ensure their actual availability in a relevant research context. To become effectively visible, a firm, persistent link from the metadata of the deposited specimen to the respective character data in a repository would be a solution. Such links can be stored and conveniently exchanged in the standard metadata exchange formats for specimens (ABCD or Darwin Core). When accessing such a specimen, e.g. via online specimen catalogues, the link to existing character data sets becomes readily available. Alternatively, the array of specimen-associated data can be extended to also include character datasets themselves. Recently, a system of persistent http-URI identifiers for collection items associated with the digital representation of a specimen was suggested by Hyam et al. (79), which immediately gained wide acceptance and has been further elaborated since (80). Using this system, the inclusion of character data into the array of specimen-associated data would make an attractive functional solution, facilitating brief, precise and convenient reference in scientific publications to a specimen with its digital image (if available), its metadata and existing character datasets. Such a solution would certainly help to increase significantly the visibility and re-usability of character datasets. Research collections are currently in a far-reaching process of transformation from curating pure analogous to sizable and complex collections of analogous and digital objects with the related datasets. Extending curation to specimen-based character data may secure research collections to play an appropriate key role in current and future research in systematic biology and thus in biodiversity assessment and analysis.

Our solution blazes a trail in systematic biology research for a streamlined process of taxon characterization and the additivity and re-usability of character data. The implementation is expected to be operational and available for download by the end of the project in December 2015. We have started to use this implementation in the integrative and dynamic approach for monographing the angiosperm order Caryophyllales (6). The current implementation has focused on various aspects of sample and data associations, while has relied on available software for the handling of morphological character data and for their aggregation from specimens to taxon characterization as well as from lower to higher taxon levels. The entire field of morphological character data aggregation, however, is waiting to become a subject of further developmental work. This concerns in particular three complexes: the modelling of character data (3); semantic web solutions for ontologies of descriptive terms (3, 81); the exchangeability of data and the interoperability of different character data matrices (e.g. merging procedures for data matrices).
